# A Rare Case of Angioleiomyoma of the Palm

**DOI:** 10.5334/jbsr.3553

**Published:** 2024-05-03

**Authors:** Karel Mercken, Sten Deschuyffeleer, Peter Matthys

**Affiliations:** 1 Department Radiology, UZ Leuven, campus Gasthuisberg, Leuven, Belgium Herestraat Leuven, Belgium; 2 Orthopedics-traumatology, Hand, wrist and elbow clinic, Europe Hospitals - St-Elisabeth site, Uccle, Brussels, Belgium; 3 Department Radiology, Europe Hospitals - St-Elisabeth site, Uccle, Brussels, Belgium

**Keywords:** Angioleiomyoma, ultrasound, MRI, hypervascular, dark reticular sign

## Abstract

*Teaching point:* Angioleiomyoma is defined on MR by a peripheral T1- and T2-hypointense rim, adjacent tortuous vascular structures, and a dark reticular sign.

## Case

**Figure 1:**
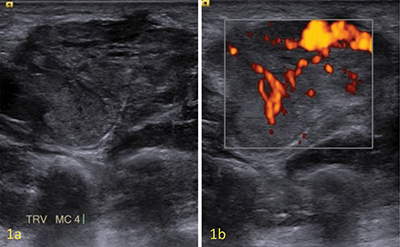
Ultrasound showing a hyopechogenic slightly heterogenous solid soft tissue mass in close contact with the flexor tendons **(a)**, with marked vascularity at Color Doppler **(b)**.

**Figure 2:**
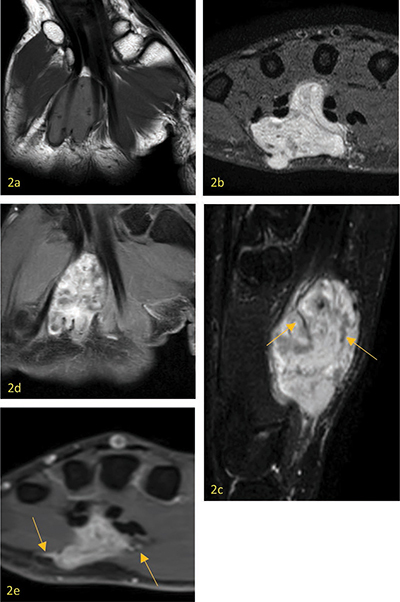
**a**. Axial T1 weighted image showing the lesion is slightly hyperintense to skeletal muscle. **b**. Coronal PD weighted image with hyperintense aspect of the lesion, with subcutaneous component. **c**. Sagittal T2 weighted image with fat suppression showing the lesion is hyperintense, with internal hypointense bandlike structures (dark reticular sign) (arrows). **d**. Axial contrast-enhanced T1 weighted image showing a strong heterogenous enhancement. **e**. Coronal contrast-enhanced T1 weighted image showing adjacent vascular structures (arrows).

**Figure 3:**
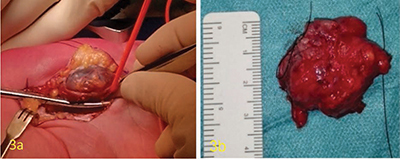
Resection piece peroperatively and postoperatively showing the lesion has the aspect of a vascular tangle.

A 45-year-old man was referred by the orthopedist for an ultrasound concerning a growing swelling in the palm with localized discomfort. Ultrasound shows a well-defined hypoechogenic slightly heterogeneous solid soft tissue mass in close contact with the flexor tendons of the third and fourth rays, extending profoundly in between them (Figure 1a). Color Doppler shows strong vascularity (Figure 1b). Magnetic resonance imaging (MRI) clearly shows a subcutaneous lesion component. The lesion is slightly T1-hyperintense compared to skeletal muscle (Figure 2a) and heterogeneously hyperintense at T2-fatsat and PD-weighted imaging (Figure 2b,c). Multiple T2-hypointense bandlike structures (dark reticular sign) can be seen (Figure 2c arrows). After intravenous Gadolinium injection, there was a strong, heterogenous enhancement of the mass (Figure 2d,e), with some adjacent vascular structures (Figure 2e arrows). After complete excision, the mass looked like a red-purple-colored vascular tangle just over 3 cm (Figure 3a,b). Microscopic examination shows benign proliferation of smooth muscle tissue associated with vascular proliferation, without cytological atypia or mitosis. Immunohistochemical examinations ruled out malignancy.

## Comment

Angioleiomyoma is a benign pericytic soft tissue lesion originating from the smooth muscle layer of blood vessels. Its etiology remains unknown. Angioleiomyoma has a broad anatomical distribution but usually affects the subcutis or dermis of extremities, most frequently the lower leg and, more rarely, the hand. Diameters range up to 4 cm. The peak incidence lies between 40 and 70 years old. Patients present with a solitary, firm, well-circumscribed, slow-growing, subcutaneous nodule or lump, which can be uncomfortable or painful. The differential diagnoses include a wide range of benign and malignant soft-tissue tumors, such as angiomyolipoma, myopericytoma, schwannoma, glomus tumor, leiomyosarcoma, and synovial sarcoma. Preoperative diagnosis is often difficult [1].

Conventional radiography could show a nonspecific soft-tissue mass or be completely normal. Acral areas may exhibit calcification, which has been considered degenerative in nature and most likely caused by repetitive mild trauma. Computed tomography (CT) shows a well-defined soft tissue mass with tissue attenuation resembling skeletal muscle. On ultrasound, a solid well-defined homogenous hypoechogenic mass is seen, with marked hypervascularity in color doppler settings (Figure 1a,b). Sometimes feeding vessels are visible. Depending on the histological subtype, echogenicity and vascularity may differ. MRI shows a well-defined mass, T1-isointense—slightly hyperintense compared to skeletal muscle (Figure 2a), and variable heterogenous T2 signal intensity (Figure 2c). Both T1- and T2-weighted images show a peripheral hypointense rim. On T2-weighted images, a dark reticular sign seems to be a hallmark of angioleiomyoma (Figure 2c arrows). Contrast-enhanced MRI can show both homogeneous and heterogeneous enhancement. Adjacent tortuous vascular structures can be seen post-contrast administration (Figure 2e arrows). [1]

Treatment consists of lesion resection.
